# Longitudinal Changes in Self-Esteem and Evaluation of Patient Satisfaction After Keratopigmentation for Unilateral Corneal Opacity

**DOI:** 10.7759/cureus.116216

**Published:** 2026-09-14

**Authors:** Rita Hepsi Rani, Thendral Velmurugan

**Affiliations:** 1 Ophthalmology, Government Medical College Thoothukudi, Thoothukudi, IND; 2 Ophthalmology, University of Virginia, Charlottesville, USA

**Keywords:** corneal opacity, corneal tattooing, cosmetic rehabilitation, keratopigmentation, patient-reported outcomes, patient satisfaction, self-esteem

## Abstract

Purpose

The primary objective of this study was to evaluate longitudinal changes in self-esteem over six months following keratopigmentation (KTP) in adults with unilateral corneal opacity and poor visual prognosis. The secondary objectives were to compare baseline self-esteem between the KTP cohort and an age- and sex-matched control group with non-disfiguring visual impairment and to examine the association between baseline self-esteem and postoperative patient satisfaction domain scores.

Methods

This prospective study included 42 adults with unilateral corneal opacity that had remained stable for at least six months and had no potential for visual recovery. The Rosenberg Self-Esteem Scale (RSES) was administered preoperatively and at one, three, and six months postoperatively, whereas the Patient Satisfaction Questionnaire-18 (PSQ-18) was administered only at the one-month postoperative visit. An age- and sex-matched control group with visual impairment but no visible ocular disfigurement was included for comparison of baseline self-esteem. Longitudinal changes in RSES scores were analyzed using the Friedman test, followed by Wilcoxon signed-rank tests for pairwise comparisons. Baseline RSES scores and postoperative PSQ-18 domain scores were compared using the Mann-Whitney U test. For the PSQ-18 analysis, participants were categorized as having low (≤15) or normal/high (>15) baseline self-esteem.

Results

RSES scores differed significantly across the four assessment points (χ²(3) = 94.39, p < 0.001) and were higher at one, three, and six months than at baseline. Baseline RSES scores were lower in the KTP cohort than in controls (10 (IQR, 9-13) vs. 20 (IQR, 18-22); p < 0.001). At one month, participants with normal/high baseline self-esteem reported higher PSQ-18 technical quality scores (Bonferroni-adjusted p = 0.020); no significant differences were observed in the other domains. No intraoperative or postoperative adverse events were observed.

Conclusion

KTP was associated with improved self-esteem over six months in this cohort. However, without a longitudinal untreated control group, the improvement cannot be attributed exclusively to KTP.

## Introduction

Globally, an estimated 6.2 million people are unilaterally blind, and 5.5 million are bilaterally blind because of corneal opacity [1]. Visible corneal scarring can affect not only appearance but also self-perception and social functioning. Individuals with facial or periocular disfigurement often report lower quality of life and self-esteem than unaffected individuals. In eyes with poor visual prognosis, keratopigmentation (KTP) offers a practical cosmetic option for masking visibly disfiguring corneal opacities.

Patient-reported outcome measures allow the effects of cosmetic procedures to be evaluated from the patient’s perspective. The Rosenberg Self-Esteem Scale (RSES) is a validated instrument for assessing global self-esteem [2], whereas the Patient Satisfaction Questionnaire-18 (PSQ-18) evaluates satisfaction with healthcare across multiple domains [3,4].

Although cosmetic interventions in plastic surgery and dermatology have been associated with improvements in self-esteem, the psychosocial effects of ocular cosmetic procedures, particularly KTP, remain underexplored.

The primary objective of this study was to evaluate longitudinal changes in self-esteem over six months following KTP in adults with unilateral corneal opacity and poor visual prognosis. The secondary objectives were to compare baseline self-esteem scores between the KTP cohort and an age- and sex-matched control group with non-disfiguring visual impairment, thereby evaluating the psychosocial burden associated with visible corneal scarring, and to examine the association between baseline self-esteem and postoperative patient satisfaction scores across the domains of the PSQ-18.

## Materials and methods

Participants

KTP Group

A total of 42 adult patients (≥18 years) with unilateral leucomatous corneal opacity, no perception of light, and no visual prognosis were recruited from the Department of Ophthalmology, Tirunelveli Medical College Hospital, between July 2023 and May 2024.

Patients with stable corneal opacity for at least six months and without signs of infection, inflammation, or epithelial breakdown were included.

Patients with bilateral corneal opacities, visible disfigurement in the fellow eye, past history of cosmetic corneal procedures, or corneal opacities requiring treatment for non-cosmetic indications (e.g., infection or pain control) were excluded. Pregnant or lactating women and individuals with a known psychiatric illness were also excluded.

*Control Group* 

During the same recruitment period, control participants were recruited from the Department of Ophthalmology, Tirunelveli Medical College Hospital. Eligible controls had non-disfiguring ocular conditions, including refractive error, early cataract, primary open-angle glaucoma, dry eye disease without corneal scarring, or central retinal vein occlusion, and had best-corrected visual acuity worse than 6/60 in the study eye. Controls were frequency-matched to the KTP cohort according to sex and the age categories (≤35, 36-50, and >50 years). Because frequency matching rather than individual matching was performed, no fixed age-matching tolerance was applied. Visual acuity was not used as a matching criterion.

The study adhered to the Declaration of Helsinki, and written informed consent was obtained from all the participants.

Sample size

No formal sample size calculation was performed because this was designed as an exploratory prospective study. Consecutive eligible patients meeting the inclusion criteria during the study period were enrolled.

Data collection 

Data were collected using a structured proforma and entered into a Microsoft Excel (Microsoft Corp., Redmond, WA, USA) spreadsheet. Preoperatively, visual acuity, intraocular pressure, and slit-lamp biomicroscopy findings were recorded. 

Patients in the KTP group underwent clinical examinations on postoperative day 1 and at one, three, and six months. The RSES was administered preoperatively and at one, three, and six months postoperatively, whereas the PSQ-18 was administered at the one-month postoperative visit. Control participants completed the RSES once at enrollment. The questionnaires were administered by an independent assessor who was not involved in the surgical procedure or clinical care and was masked to the participants’ previous questionnaire scores. Both questionnaires were interviewer-administered in Tamil using translated versions of the original instruments. At each postoperative visit, the operated eyes were examined for potential complications, including corneal perforation, pigment extrusion, color fading, corneal edema, progression of scarring, pigment-related toxic reactions, microbial keratitis, changes in intraocular pressure, and corneal neovascularization.

Surgical technique

All procedures were performed by a single experienced corneal surgeon under local anesthesia using manual intrastromal KTP. A 2-mm corneal entry was created, followed by manual dissection of a lamellar stromal tunnel at a standardized depth of 150 µm using a crescent blade and corneal spatula. The same instruments, entry size, and stromal depth were used in all cases, whereas the extent of the pocket was adjusted to cover the corneal opacity. A commercially available, sterile, premixed black tattoo ink (Dynamic Black; Dynamic Color Company, Fort Lauderdale, FL, USA) was introduced incrementally into the stromal pocket and distributed uniformly over the stromal bed. According to the manufacturer’s safety data sheet, the formulation contained 10%-15% w/w carbon black (C.I. 77266), 6%-12% w/w isopropyl alcohol, 1%-6% w/w acrylic polymer, and water as the balance. The amount of pigment was adjusted according to the extent of the opacity and injected until uniform pigmentation was achieved.

Rosenberg Self-Esteem Scale (RSES) 

The RSES is a validated 10-item questionnaire, with scores ranging from 0 to 30; higher scores indicate greater self-esteem. We assessed RSES scores preoperatively and at one, three, and six months postoperatively [2].

Patient Satisfaction Questionnaire (PSQ-18)

Patient satisfaction was measured using the PSQ-18, which has domain scores for general satisfaction, technical quality, interpersonal manner, communication, financial aspects, time with doctor, and accessibility. The standard scoring system was applied [3].

Statistical analysis

Data were analyzed using Stata version 18.0 (StataCorp LLC, College Station, TX, USA). Continuous variables were presented as means with standard deviations, and categorical variables as frequencies and percentages. Changes in RSES scores were analyzed using the Friedman test to compare preoperative scores with postoperative scores at one month, three months, and six months, followed by Wilcoxon signed-rank tests with Bonferroni correction for pairwise comparisons. PSQ-18 scores were compared between participants with low (≤15) and normal/high (>15) baseline self-esteem using the Mann-Whitney U test. To account for testing across the seven PSQ-18 domains, Bonferroni-adjusted p-values were calculated. A two-tailed p-value < 0.05 was considered statistically significant.

## Results

A total of 42 patients (42 eyes) underwent KTP, and 36 participants were included in the control group. The mean age was 35.2 ± 7.09 years in the KTP group and 38.09 ± 9.02 years in the control group. The median baseline RSES score was significantly lower in the KTP group than in the control group (10 (IQR 9-13) versus 20 (IQR 18-22); Mann-Whitney U = 94.5, z = -6.656, p < 0.001). Trauma and graft failure were the leading causes of corneal opacity, followed by congenital and postoperative causes. The baseline demographic and clinical characteristics are presented in Table 1.

**Table 1:** Baseline demographic and clinical characteristics of the keratopigmentation and control groups Categorical variables are presented as n (%), age is additionally presented as mean ± standard deviation (SD), and baseline RSES scores are presented as median (interquartile range, IQR). Control participants were age- and sex-matched to the KTP group. KTP: keratopigmentation, RSES: Rosenberg Self-Esteem Scale.

Characteristic	Category/measure	Control (n = 36)	KTP (n = 42)
Age	Mean ± SD, years	38.09 ± 9.02	35.20 ± 7.09	
	≤35 years	15 (41.67%)	20 (47.6%)
	36-50 years	16 (44.44%)	20 (47.6%)
	>50 years	5 (13.89%)	2 (4.8%)
Sex	Male	15 (41.67%)	18 (42.9%)
	Female	21 (58.33%)	24 (57.1%)
Eye	Left	20 (55.56%)	17 (40.5%)
	Right	16 (44.44%)	25 (59.5%)
Duration	≤1 year	17 (47.22%)	20 (47.6%)
	>1 year	19 (52.78%)	22 (52.4%)
Baseline RSES score	Median (IQR)	20 (18-22)	10 (9-13)

RSES scores differed significantly across the four study visits (Friedman test: χ²(3) = 94.39, p < 0.001). Pairwise Wilcoxon signed-rank tests with Bonferroni correction showed significantly higher RSES scores at one, three, and six months compared with baseline (all p < 0.001) (Table 2).

**Table 2:** Changes in Rosenberg Self-Esteem Scale (RSES) scores following keratopigmentation Overall differences across study visits were analyzed using the Friedman test. Pairwise comparisons between baseline and one, three, and six months were performed using Wilcoxon signed-rank tests with Bonferroni correction.

Test	Comparison	n	Statistic (χ² or z)	p-value
Friedman test (overall)	Pre-op, 1 month, 3 months, and 6 months	42	χ²(3) = 94.39	<0.001
Wilcoxon signed-rank tests (pairwise)	Pre-op vs 1 month	42	z = -5.650	<0.001
	Pre-op vs 3 months	42	z = -5.651	<0.001
	Pre-op vs 6 months	42	z = -5.651	<0.001

PSQ-18 domain scores were compared between participants with low (≤15) and normal/high (>15) baseline self-esteem. Participants with normal/high baseline self-esteem had significantly higher technical quality scores than those with low baseline self-esteem (p = 0.020). No statistically significant differences were observed in general satisfaction, interpersonal manner, communication, financial aspects, time spent with the doctor, or accessibility and convenience (Table 3).

**Table 3:** Comparison of PSQ-18 domain scores at the one-month postoperative visit between participants with low and normal/high baseline self-esteem Values are presented as median (interquartile range, IQR). p-values were calculated using the Mann-Whitney U test, comparing participants with low (≤15) and normal/high (>15) baseline self-esteem. A p-value <0.05 was considered statistically significant. PSQ-18: Patient Satisfaction Questionnaire-18.

PSQ-18 domain	Low self-esteem (≤15), median (IQR)	Normal/high self-esteem (>15), median (IQR)	p-value (Mann–Whitney U)
General satisfaction (GS)	4.00 (4.00-5.00)	5.00 (4.50-5.00)	0.168
Technical quality (TQ)	4.00 (3.00-4.00)	5.00 (4.50-5.00)	0.020
Interpersonal manner (IM)	4.00 (3.00-5.00)	4.00 (3.00-5.00)	0.890
Communication (Co)	4.00 (4.00-5.00)	4.00 (4.00-4.50)	0.453
Financial aspects (FA)	4.00 (4.00-5.00)	4.50 (4.00-5.00)	0.698
Time spent with doctor (TS)	4.00 (3.00-5.00)	3.50 (3.00-4.50)	0.616
Accessibility and convenience (AC)	4.00 (4.00-5.00)	4.00 (4.00-4.50)	0.669

No intraoperative complications or postoperative adverse events were observed during the six-month follow-up period.

Figure 1 illustrates the key steps of the KTP procedure, including stromal-pocket creation and dye placement. Figure 2 presents representative preoperative and postoperative photographs demonstrating improved cosmetic appearance and symmetry between the eyes.

**Figure 1:**
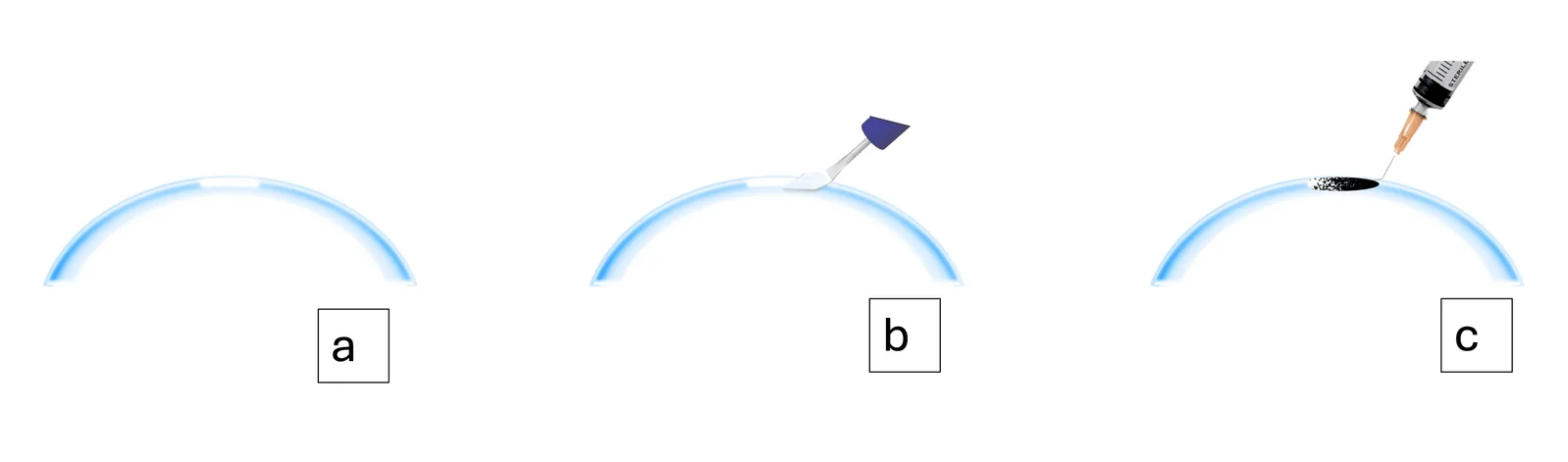
Schematic illustration of the key steps involved in the keratopigmentation (KTP) procedure (a) Corneal cross-section demonstrating a central stromal opacity. (b) Creation of a stromal pocket using a crescent blade, followed by stromal dissection with a corneal spatula. (c) Injection of pigment dye into the stromal pocket to achieve uniform corneal pigmentation. Image Credit: This figure was created using Adobe Photoshop (Adobe, San Jose, CA, USA).

**Figure 2:**
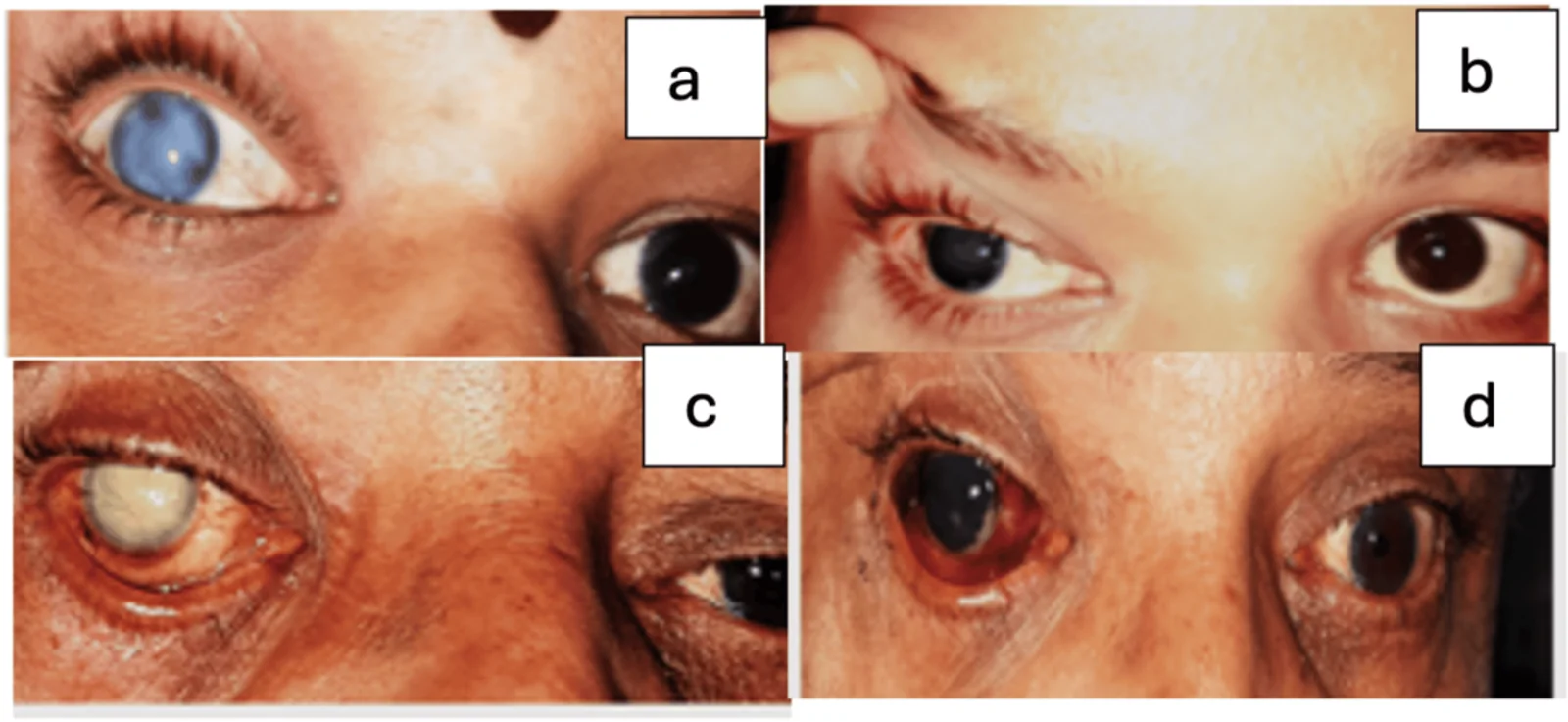
Representative preoperative and postoperative clinical photographs of two patients who underwent keratopigmentation (KTP) (a) Preoperative appearance of Patient 1 with cosmetically significant unilateral corneal opacity. (b) Postoperative appearance of Patient 1 following KTP. (c) Preoperative appearance of Patient 2 with cosmetically significant unilateral corneal opacity. (d) Postoperative appearance of Patient 2 following KTP. Note: Written informed consent to include these images in an open-access article was obtained from the patients.

## Discussion

The eyes are often the first feature noticed during interpersonal communication, and visible ocular disfigurement may affect self-perception and social interaction. In this study, RSES scores increased at one month after KTP and remained higher than baseline at three and six months. These findings indicate that KTP was associated with sustained improvement in self-esteem scores over the six-month follow-up period. Nearly half of the participants were between 36 and 50 years of age. Trauma and failed corneal grafts were the leading causes of cosmetically significant unilateral corneal opacity, consistent with previous reports from India identifying post-traumatic corneal scarring as a major cause of unilateral ocular disfigurement [5].

Baseline self-esteem scores were significantly lower among patients with visible corneal opacity than among control participants with visual impairment but no visible corneal opacity. This finding suggests an association between visible ocular disfigurement and lower self-esteem beyond that associated with visual impairment alone. This interpretation is consistent with a large population-based study by Maaswinkel et al., who showed that self-esteem and a sense of mastery mediated the association between reduced visual acuity and poor mental health [6].

This observation fits within a broader body of literature linking eye disease to psychological well-being. An individual's self-esteem is an enduring inner belief system that helps define their personality and guide interpersonal interactions [6]. For many years, researchers have examined the potential mental health effects of eye disease. They have investigated whether vision-related issues may affect people's overall moods, happiness, and/or anxiety. Yamanishi et al. reported that patients diagnosed with glaucoma had significantly lower subjective happiness than those without ocular disease, regardless of the presence or absence of visible disability [7]. Furthermore, a meta-analysis by Parravano et al. found that approximately one in every four visually impaired patients suffered from clinical depression [8], while Senra et al., similarly, demonstrated that in patients with chronic eye disease, anxiety modified the association between visual acuity and quality of life, emphasizing the psychosocial burden of vision loss [9].

Disfiguring ocular lesions can deeply influence how individuals perceive themselves and interact socially. Poor self-esteem is often linked to depression, social withdrawal, and a reduced quality of life [10-12]. Our results align with these observations, suggesting that aesthetic rehabilitation of the eye may be associated with improved self-esteem and psychosocial well-being. Improved appearance may contribute to enhanced mood, self-confidence, and interpersonal relationships, as supported by earlier studies on the positive link between physical self-image and psychological health [13,14].

Although the psychological impact of vision loss is well established, few studies have specifically examined the improvement in self-esteem following correction of corneal disfigurement. Our study helps bridge this gap by demonstrating sustained increases in RSES scores over six months following KTP; however, these findings represent an association and do not establish a causal effect of the procedure.

In the broader context of aesthetic surgery, improving confidence and social comfort represents an important treatment objective. Alotaibi et al. observed that while motivations and acceptance of cosmetic procedures vary across populations, the underlying pursuit of confidence and social belonging is universal [15].

In the PSQ-18 analysis, the most notable finding was observed in the technical quality domain, for which participants with normal/high baseline self-esteem reported significantly higher scores than those with low baseline self-esteem. Although differences in the other PSQ-18 domains did not reach statistical significance, the findings suggest that baseline self-esteem may be associated particularly with patients’ perceptions of the technical quality of care.

All procedures were performed by a single experienced corneal surgeon, minimizing variability in surgical technique and perioperative counselling. Although this standardized care within the study, it may limit the generalizability of the findings to other surgeons and clinical settings.

The present findings have important clinical implications. KTP was associated with higher postoperative self-esteem scores in this cohort. However, larger controlled studies are required to determine whether these improvements are attributable to the procedure and whether they persist beyond six months.

Limitations

The study was conducted at a single center with a relatively small sample size and a six-month follow-up. As this was an exploratory prospective study, no formal a priori sample size calculation was performed. Furthermore, the absence of a non-intervention comparison group means that placebo effects, expectation bias, and the Hawthorne effect cannot be excluded.

## Conclusions

KTP was associated with a significant improvement in self-esteem among individuals with cosmetically significant unilateral corneal opacity and poor visual prognosis. Participants also reported high levels of satisfaction with their care, with technical quality demonstrating the strongest association with baseline self-esteem. These findings suggest that, beyond improving corneal appearance, KTP may be associated with a reduced psychosocial burden from visible corneal scarring and enhanced self-esteem and confidence in appropriately selected patients. The use of validated patient-reported outcome measures highlights the importance of incorporating psychosocial outcomes into the evaluation of cosmetic ocular procedures. Larger multicenter studies with longer follow-up and appropriate comparison groups are warranted to confirm these findings and evaluate their long-term sustainability.
